# Potential Mechanisms of SGLT2 Inhibitors for the Treatment of Heart Failure With Preserved Ejection Fraction

**DOI:** 10.3389/fphys.2021.752370

**Published:** 2021-11-05

**Authors:** Steffen Pabel, Nazha Hamdani, Jagdeep Singh, Samuel Sossalla

**Affiliations:** ^1^Department of Internal Medicine II, University Hospital Regensburg, Regensburg, Germany; ^2^Department of Molecular and Experimental Cardiology, Institut für Forschung und Lehre (IFL), Ruhr University Bochum, Bochum, Germany; ^3^Department of Cardiology, St. Josef-Hospital, Ruhr University Bochum, Bochum, Germany; ^4^The Heart Centre, Royal Infirmary of Edinburgh, Edinburgh, United Kingdom; ^5^Clinic for Cardiology and Pneumology, Georg-August University Göttingen, DZHK (German Centre for Cardiovascular Research), Partner Site Göttingen, Göttingen, Germany

**Keywords:** heart failure, HFpEF—heart failure with preserved ejection fraction, SGLT2 inhibitors, diastolic function, inflammation, oxidative stress

## Abstract

Heart failure with preserved ejection fraction (HFpEF) is an unsolved and growing concern in cardiovascular medicine. While no treatment options that improve prognosis in HFpEF patients has been established so far, SGLT2 inhibitors (SGLT2i) are currently being investigated for the treatment of HFpEF patients. SGLT2i have already been shown to mitigate comorbidities associated with HFpEF such as type 2 diabetes and chronic renal disease, however, more recently there has been evidence that they may also directly improve diastolic function. In this article, we discuss some potential beneficial mechanisms of SGLT2i in the pathophysiology of HFpEF with focus on contractile function.

## Introduction: Heart Failure With Preserved Ejection Fraction—An Unmet Clinical Need

Heart failure (HF) with preserved ejection fraction (HFpEF) is diagnosed in a growing proportion of patients presenting with symptoms of HF (Redfield, 2016). Patients with HFpEF are characterized by clinical signs of HF with evidence of diastolic dysfunction, while systolic function is preserved. Clinical data indicate that morbidity and mortality in HFpEF patients is comparable to those with HFrEF (Redfield, 2016). Until very recently, in contrast to HFrEF, no prognostically relevant treatment strategy could be established for HFpEF patients; many efficacious drugs used in HFrEF failed to improve prognosis in HFpEF patients. Current and future pharmacological endeavors face the difficulties of a highly heterogenous HFpEF population, involving a variety of comorbidities and pathomechanisms. While HFpEF is a complex and multifaceted disease, different effects of sodium-glucose-cotransporter 2 inhibitors (SGLT2i) on mechanisms considered to be involved in HFpEF pathophysiology have been reported. Very recently, the EMPEROR-Preserved trial was the first positive study reporting a reduction of the combined risk of cardiovascular death or hospitalization for HF in patients with HFpEF after treatment with empagliflozin (Anker et al., 2021a). This review will discuss the potential mechanisms of SGLT2i in HFpEF patients, paving the way for a novel pharmacological option for HFpEF patients.

## Clinical Evidence of SGLT2 Inhibitors for the Treatment of Heart Failure With Preserved Ejection Fraction Patients

SGLT2i were initially used as oral anti-diabetes agents via blood glucose reduction from the inhibition of SGLT2 transporters in the kidney. Remarkably, SGLT2i showed distinct beneficial effects on cardiovascular outcomes in patients with type 2 diabetes mellitus (T2DM) but also in patients with HF independent of T2DM (Zinman et al., 2015; Neal et al., 2017; McMurray et al., 2019; Wiviott et al., 2019; Packer et al., 2020). The DAPA-HF trial was the first phase 3, placebo-controlled trial, which randomly assigned 4744 patients with New York Heart Association class II to IV HF with an ejection fraction of 40% or less to receive either dapagliflozin or placebo, on top of guideline recommended therapy. The primary outcome, a composite of worsening HF or cardiovascular death, was significantly and remarkably reduced in patients treated with dapagliflozin (McMurray et al., 2019). Similarly, empagliflozin significantly diminished cardiovascular death and hospitalization for HF in patients with HFrEF (Packer et al., 2020). Importantly, these improved hard outcomes in HF patients were independent of T2DM in both trials (McMurray et al., 2019; Packer et al., 2020; Petrie et al., 2020; Anker et al., 2021b). This has led to the redefinition of the pharmacological landscape in HFrEF, with SGLT2i now being recommended by clinical guidelines for HF (Cosentino et al., 2020; McDonagh et al., 2021; Writing et al., 2021).

Given the favorable cardiovascular outcomes of SGLT2i in patients with T2DM and established cardiovascular disease or at high risk and in patients with HFrEF, SGLT2i are currently being investigated in HFpEF. The recently published EMPEROR-Preserved trial investigated the effect of empagliflozin on the composite endpoint of cardiovascular death or HF hospitalization in 5988 patients with HF and an EF > 40% (NYHA II-IV, elevated NT-proBNP, structural heart disease or HF hospitalization). Empagliflozin significantly reduced the primary endpoint, which was mainly driven by a ∼29% reduced risk of hospitalization for HF (Anker et al., 2021a). Although the effect was more pronounced in patients with mildly reduced EF, the effect was still present up to an EF of < 60%. Therefore, SGLT2i showed to be the first evidence-proved drug for these respective patients in the absence of HFrEF. In addition to the EMPEROR-Preserved trial, the randomized controlled DELIVER trial is studying the effects of dapagliflozin on cardiovascular death or HF events in patients with HFpEF (EF > 40%, structural heart disease, Elevated NT-pro BNP levels, NYHA II-IV). Both these trials undoubtedly define the role of SGLT2i in HFpEF. Of note, it has to be mentioned that the inclusion criteria of an EF > 40% in both clinical trials lack the typical HFpEF definition (preserved EF). This has, however, to be discussed elsewhere. Additionally, there has been some previous evidence pointing toward favorable effects of SGLT2i in HFpEF patients.

A recent meta-analysis of randomized controlled studies regarding effects of SGLT2i in ∼16,000 patients with HF with or without T2DM, indicated that the subgroup of patients with HFpEF may also achieve a risk reduction of the composite endpoint of cardiovascular death or HF hospitalization (Singh et al., 2021). The SOLOIST-WHF trial investigated the effects of the dual SGLT1 and SGLT2 inhibitor sotagliflozin in 1222 T2DM patients recently hospitalized for worsening of HF. The investigation of sotagliflozin is of particular interest as it also inhibits SGLT1, which is, in contrast to SGLT2, expressed in the myocardium (Di Franco et al., 2017). Sotagliflozin reduced the composite of cardiovascular death, HF hospitalization and urgent HF visit consistently across different subgroups including patients with an EF > 50% (Bhatt et al., 2021). Whereas pooled data from the SCORED and the SOLOIST-WHF trial showed a reduction in the composite of cardiovascular death, HF hospitalization and urgent HF visit after treatment with sotagliflozin compared to placebo (Bhatt et al., 2021), in 739 patients with HFpEF (EF > 50%). Another exploratory analysis of the data from the DECLARE-TMI 58 trial, the VERTIS CV and pooled data from the SOLOIST−WHF and the SCORED study also suggests favorable effects SGLT2i on the composite of HF hospitalization and cardiovascular death in patients with HFpEF (Butler et al., 2020). Moreover, one could assume in the early cardiovascular outcome trials of SGLT2i in patients with T2DM (i.e., EMPA-REG Outcome trial, CANVAS trial, DECLARE-TIMI 58 trial), a substantial proportion of patients may have had undiagnosed HFpEF due to the comorbidities and risk profile of the trial participants (Zinman et al., 2015; Neal et al., 2017; Wiviott et al., 2019). Finally, existing evidence of the effects of SGLT2i on comorbidities relevant to HFpEF pathophysiology lends credence to the investigation of SGLT2i in HFpEF.

## Effects of Sodium-Glucose-Cotransporter 2 Inhibitors on Heart Failure With Preserved Ejection Comorbidities: Cutting the Roots Instead of Cutting the Tree?

Inhibition of SGLT2 transporters in the kidney causes glucosuria, natriuresis, and osmotic diuresis. This results in lower blood glucose levels, obesity, blood pressure and improved lipid metabolism (Abdul-Ghani et al., 2016; Mancia et al., 2016; Benham et al., 2021). All of these are typical comorbidities in HFpEF patients and are associated with increased morbidity and mortality in HFpEF (Mentz et al., 2014). Therefore, it is tempting to speculate that SGLT2i may be beneficial in HFpEF patients because their pleiotropic effects target the multifaceted pathophysiology of HFpEF.

However, some arguments against a major contribution of classical cardiovascular risk factors for the improvement of clinical outcomes should be discussed. It has been suggested that at least in diabetic patients the reduction of cardiovascular risk factors like blood pressure (Benham et al., 2021), cholesterol (Langslet et al., 2020), or blood glucose (Fitchett et al., 2017, 2018, 2019) are unlikely to be responsible for the prognostic benefits seen with SGLT2i. It is also known that improvement of atherosclerotic risk is not considered as main mechanism to improve prognosis in diabetic patients (Fitchett et al., 2019; Zelniker et al., 2019; Arnott et al., 2020). This is also supported by the early time course of the prognostic effects of SGLT2i in clinical trials in patients with T2DM and high cardiovascular risk (Verma et al., 2017; Berg et al., 2021). However, a contribution to the later separation of the curves cannot be ruled out.

Another important consideration is cardiorenal syndrome: The hallmark feature of HF is salt and water retention, both of which are regulated by the kidneys, therefore the intimate interaction between the heart and kidneys cannot be discounted. The Study to Evaluate the Effect of Dapagliflozin on Renal Outcomes and Cardiovascular Mortality in Patients With Chronic Kidney Disease (Dapa-CKD) and Evaluation of the Effects of Canagliflozin on Renal and Cardiovascular Outcomes in Participants With Diabetic Nephropathy (CREDENCE) trials both showed striking benefits in hard renal outcomes including progression of CKD, end stage renal disease and death (Perkovic et al., 2019; Heerspink et al., 2020). Interestingly, even in these “renal outcome trials” the benefits to HF outcomes remained very robust. The putative mechanism of renal benefits stem from a reduction in trans-glomerular pressure, thereby preserving glomerular longevity. This results from renal afferent arteriolar vasoconstriction due to tubuloglomerular feedback from increased sodium delivery to the macula densa following inhibition of the SGLT2 transporter (Lytvyn et al., 2017). It follows that preservation of renal function will have salutary cardiac effects given the kidneys are the downstream target organ of the natriuretic peptide and renin-angiotensin-aldosterone systems, which play critical roles in HF.

It is known that patients with either clinically stable HFpEF, or at hospital admission due to HFpEF, worsening of renal function is independently associated with all-cause mortality (Roth et al., 2017; Kang et al., 2018). Taken together, renoprotection is therefore very likely one of the key extra-cardiac benefits of SGLT2i therapy which may lead to improved outcomes in HFpEF patients.

## Effects of Sodium-Glucose-Cotransporter 2 Inhibitors on Diastolic Function

HFpEF on the myocardial level is characterized by diastolic dysfunction with impaired relaxation leading to compromised filling of the ventricles. Thus, improving diastolic function should theoretically be the ultimate treatment strategy for patients with HFpEF. While there is currently no approved therapy for the specific treatment of diastolic dysfunction, there is growing evidence suggesting that SGLT2i may directly target diastolic function. In small but prospective clinical studies in patients with T2DM and normal EF, SGLT2i improved diastolic function as determined by echocardiography after 3 (Matsutani et al., 2018) and 6 months of treatment (Shim et al., 2021). Likewise, in another small prospective but uncontrolled trial in patients with T2DM and high atherosclerotic risk, an improvement of diastolic function after 3 months empagliflozin treatment was reported (Verma et al., 2016). These clinical findings are supported by experimental evidence; in different diabetic mice models chronic treatment with empagliflozin mitigated diastolic dysfunction as measured by echocardiography (Habibi et al., 2017; Hammoudi et al., 2017) or pressure catheter (Moellmann et al., 2020). Of note, SGLT2i-treated and untreated diabetic animal models might have higher differences in blood glucose levels depending on the treatment with SGLT2i compared to patients in clinical trials with already established antidiabetic therapy. In obese diabetic rats that are characterized by diastolic dysfunction, treatment with empagliflozin acutely shortened isovolumetric relaxation time and increased the E/A ratio indicating improved diastolic function (Pabel et al., 2018). Notably, also in a DOCA-salt induced rodent HFpEF model, empagliflozin improved pathological diastolic parameters and relaxation measured by echocardiography and pressure-volume loops (Connelly et al., 2019).

As SGLT2i have broad systemic effects, investigations excluding different confounders are needed to further clarify the effects of SGLT2i on diastolic function. Our group provided first evidence of favorable effects of empagliflozin on diastolic function in human ventricular trabecula from patients with HFrEF (Pabel et al., 2018). Empagliflozin acutely mitigated pathological diastolic stiffness in the human specimens. The study firstly showed that these effects were independent of T2DM. Importantly, the human trabeculae were studied *in vitro*, in the absence of systemic confounders (e.g., alterations of blood pressure or volume shift) as may occur in any *in vivo* model. Thus, these experiments indicate a direct cardiac effect of SGLT2i-induced improvement of diastolic function (Pabel et al., 2018).

To understand the potential mechanism of action of SGLT2i-induced improvement in diastolic function, one must appreciate that diastolic function is determined by (1) the myocardial stiffness based on the viscoelastic properties mediated largely by myofilament stiffness as well as structural remodeling of the extracellular matrix and (2) myocardial relaxation mediated by Ca^2+^ dissociation from troponin C and reuptake into the sarcoplasmic reticulum (Franssen and González Miqueo, 2016). The following paragraphs will discuss the potential effects of SGLT2i on these different aspects of diastolic function and their pathophysiological implications in HFpEF.

### Effects of Sodium-Glucose-Cotransporter 2 Inhibitors on Myofilament Function

**FIGURE 1:**
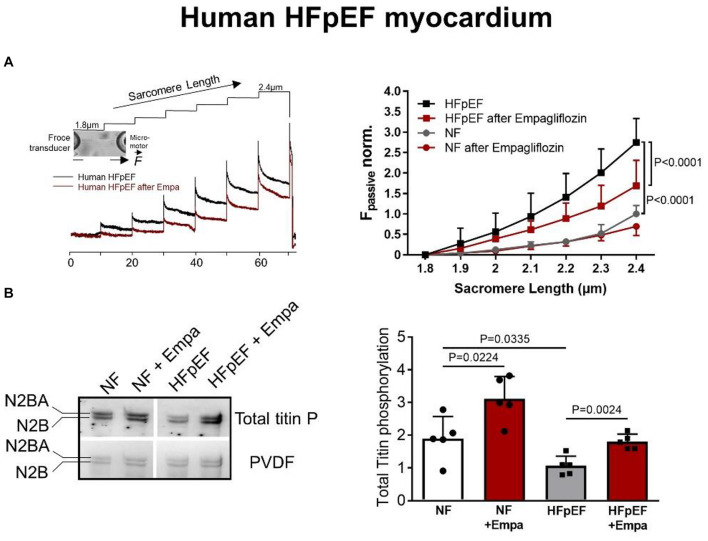
**(A)** Force response to stepwise cardiomyocyte stretches showing the effects of empagliflozin on passive myofilament stiffness of human skinned cardiomyocytes from HFpEF patients or controls (non-failing, NF) and **(B)** phosphorylation levels of titin in human HFpEF myocardium and controls ± empagliflozin treatment (Pabel et al., 2018); with permission.

**FIGURE 2:**
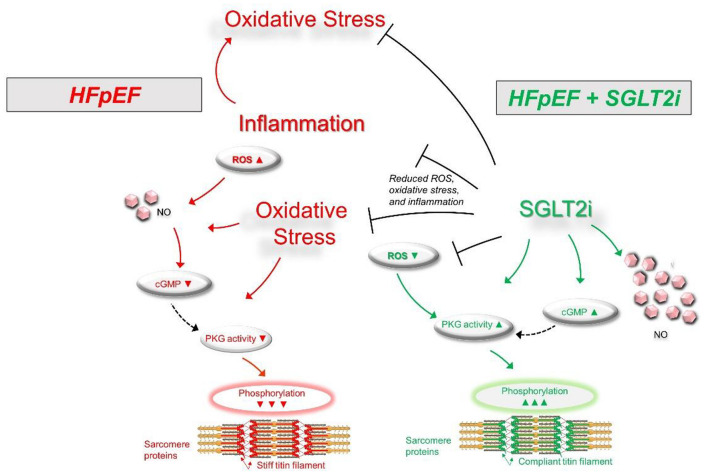
Scheme for the signaling pathways in HFpEF (left) showing an oxidative condition with impaired signaling pathways via increased inflammation and oxidative stress (Upward arrows = increase; downward arrows = decrease). SGLT2i treatment (right) may result in a significant improvement of signaling pathways via decreased inflammation and oxidative stress. cGMP, cyclic guanosine monophosphate; HFpEF, heart failure with preserved ejection fraction; NO, nitric oxide; PKG, protein kinase G; ROS, reactive oxygen species.

Myofilament function critically determines diastolic cardiomyocyte stiffness, and myofilament stiffness is abnormally increased in HFpEF patients (Borbely et al., 2005). The giant elastic protein titin is known to influence passive stiffness via isoform shift (N2BA/N2B ratio) and posttranslational modifications such as oxidation and/or phosphorylation (Linke and Hamdani, 2014). In animal and human HFpEF myocardia, altered phosphorylation of titin and other small regulatory myofilament proteins have been shown to increase passive cardiomyocyte stiffness (Hamdani et al., 2013a,b, c; Linke and Hamdani, 2014). Our previous findings demonstrated that in human HFpEF myocardium, empagliflozin restores the pathologically altered phosphorylation of titin (Figure 1) and the small regulatory proteins troponin I and myosin binding protein C (Pabel et al., 2018). Consequently, empagliflozin treatment led to a reduction of pathological cardiomyocyte stiffness in human HFpEF myocardium (Pabel et al., 2018). Furthermore, we revealed that these observations were mediated by an improvement of cyclic guanosine monophosphate (cGMP)-dependent protein kinase or protein kinase G (PKG) signaling, which is typically diminished in HFpEF myocardium and is known to underlie diastolic stiffness in HFpEF (Paulus and Tschöpe, 2013). Accordingly, the improvement of cGMP pool, which regulates PKG activity, is thus considered as a potential therapeutic target in HFpEF (Greene et al., 2013). Interestingly, empagliflozin enhanced the NO-cGMP-PKG pathway after 8 weeks of treatment in diabetic mice (Xue et al., 2019). In human and rodent HFpEF myocardium we showed that nitric oxide bioavailability increased upon acute empagliflozin treatment resulting in elevated cGMP levels and increased PKG activity. As a consequence, PKG-dependent phosphorylation of myofilament proteins was restored (Kolijn et al., 2021). Recently, in pigs with myocardial infarction induced HFrEF 2 months of treatment with empagliflozin also resulted in an improved diastolic function in invasive and non-invasive analyses, which was associated with increased NO availability and PKG signaling (Santos-Gallego et al., 2021). As PKG is centrally involved in HFpEF pathophysiology, the impact of SGLT2i on PKG signaling and myofilament function could therefore be a key-stone effect in improving diastolic function in HFpEF hearts (Figure 2), thereby resulting in material change the disease trajectory (Pabel et al., 2020a).

### Effects of Sodium-Glucose-Cotransporter 2 Inhibitors on Myocardial Fibrosis

Increased cardiac fibrosis adversely affects diastolic function and is a common feature in HFpEF patients (Zile et al., 2015). The etiology of fibrosis is heterogenous and the development of fibrotic tissue presumably takes place at later disease stages (Sweeney et al., 2020). Increased fibrosis reduces myocardial compliance thereby limiting diastolic filling (de Souza, 2002; Kasner et al., 2011; Sweeney et al., 2020). Limited evidence of the impact of SGLT2i on myocardial fibrosis has been reported. In a hypertensive HF model, 12 weeks of treatment with empagliflozin resulted in reduced cardiac remodeling with less atrial as well as ventricular fibrosis (Lee et al., 2019). In another rat model of myocardial infarction, dapagliflozin reduced myofibroblast and macrophages infiltration and thereby demonstrating antifibrotic properties (Lee et al., 2017). Reduced fibrotic content upon SGLT2i treatment was also observed in diabetic (Li et al., 2019) and afterload induced HFrEF mice (Shi et al., 2019). A possible mechanism has been provided in a diabetic mouse model, where dapagliflozin reduced myocardial fibrosis and proinflammatory markers, which was associated with regulation of AMPK (Ye et al., 2017). Accordingly, dapagliflozin increased AMPK phosphorylation in cardiac fibroblasts, which also resulted in a reduction of NHE1 mRNA expression (Ye et al., 2018).

Preventing the development of cardiac fibrosis using SGLT2i likely impedes the progression of myocardial stiffness and may therefore be advantageous for the causal treatment HFpEF patients. Nevertheless, evidence of these specific and direct mechanisms of SGLT2i on fibrosis is limited. Moreover, the effect of these agents on myocardial fibrosis may be confounded by their other cardiovascular effects which may deter disease progression via different mechanisms.

### Effects of Sodium-Glucose-Cotransporter 2 Inhibitors on Cardiac Hypertrophy

In patients presenting with HFpEF, diastolic dysfunction and cardiac hypertrophy are often concomitantly found. While the interaction of hypertrophy, diastolic function and HFpEF is complex, increased left ventricular hypertrophy impairs chamber geometry and may induce diastolic dysfunction *per se* (Heinzel et al., 2015). Along with the SGLT2i-induced improvement of diastolic function in different patient populations, an impact of SGLT2i on left ventricular hypertrophy has been demonstrated. Clinical data of a randomized placebo-controlled trial of 97 patients with preserved EF and T2DM as well as coronary artery disease demonstrated using cardiac MRI that empagliflozin reduced LV mass index after 6 months of treatment (Verma et al., 2019). A reduction of LV mass following SGLT2i therapy was also observed in echocardiographic measurements of patients with T2DM (Verma et al., 2016; Brown et al., 2020). However, in these clinical trials it is impossible to separate direct cardiac effects from secondary mechanisms such as changes of blood pressure or pre- and afterload. As of now, the mechanisms of the regression in hypertrophy warrants further investigation, in particular in HFpEF patients.

### Effects of Sodium-Glucose-Cotransporter 2 Inhibitors on Cardiomyocyte Na^+^ and Ca^2+^ Homeostasis

Cardiomyocyte Ca^2+^ homeostasis mediates excitation-contraction coupling, thereby determining myocardial contraction and relaxation. During diastole cytosolic Ca^2+^ moves back into the sarcoplasmic reticulum via the SERCA2a transporter which is modulated by phospholamban. Ca^2+^ is also eliminated from the cardiomyocyte via the Na^+^/Ca^2+^ exchanger. As cytosolic Ca^2+^ levels decrease, the passive dissociation from Troponin C changes tropomyosin conformation resulting in myocardial relaxation (Bers, 2002). Thus, Ca^2+^ homeostasis plays a critical role for diastolic function. However, data on Ca^2+^ handling in HFpEF are scarce due to limited availability of human samples and limitations of HFpEF-like animal models. Reports from HFpEF-like animal models (i.e., via age, metabolic disorders or transverse aortic constriction) indicate that systolic Ca^2+^ release and cell shortening could be unaltered or increased, while Ca^2+^ reuptake and relaxation may be impaired along with elevated diastolic Ca^2+^ depending on the model studied (Peana and Domeier, 2017; Frisk et al., 2021). SGLT2i have been reported to influence cardiomyocyte Na^+^ homeostasis and thereby Ca^2+^ handling (firstly reported by Baartscheer et al., 2017), thus potentially modulating diastolic function. In failing ventricular murine and human cardiomyocytes treated *in vitro* with empagliflozin (24 h) Ca^2+^/calmodulin-dependent protein kinase IIδ (CaMKII) activity has been found to be diminished (Mustroph et al., 2018). Consequently, aberrant diastolic sarcoplasmic reticulum Ca^2+^ leak, which elevates cytosolic Ca^2+^ levels and thereby adversely increasing diastolic tension (Fischer et al., 2013) was reduced after exposure to empagliflozin (Mustroph et al., 2018). It has therefore been speculated that this mechanism could also be involved in HFpEF pathophysiology (Eisner et al., 2020). Interestingly, in obese diabetic mice, the improvement of diastolic function was associated with an increased phospholamban phosphorylation and thus SERCA2a activity (Hammoudi et al., 2017). However, the effects of SGLT2i on cardiomyocyte Ca^2+^ homeostasis are in part controversial and difficult to interpret as experimental protocols and (disease) models varied. In human cardiomyocytes from patients with HFrEF acute treatment with empagliflozin did not change systolic Ca^2+^ transient or diastolic cytosolic Ca^2+^ (Pabel et al., 2018). Likewise, we performed a blinded experimental long-term study of human induced pluripotent stem cell cardiomyocytes from healthy subjects as clinical effects occur after weeks or months which is in contrast to many experimental study designs where acute exposure to these drugs have been performed. In our respective study 2 months of treatment with empagliflozin showed no impact on Ca^2+^ homeostasis and EC-coupling proteins (Pabel et al., 2020b). In a study based on Dahl salt-sensitive rats with high-salt diet serving as a HFpEF model, the authors reported that dapagliflozin beneficially affects Ca^2+^ and Na^+^ overload after *in vivo* treatment but not after direct treatment of cardiomyocytes (Cappetta et al., 2020). Therefore, also non-cardiomyocyte targets might be involved in possible effects of SGLT2i on EC-coupling.

Besides Ca^2+^ cycling, Na^+^ homeostasis has shown to influence diastolic function. As an increased Na^+^ influx is counterbalanced via Na^+^/Ca^2+^ exchanger, mechanisms elevating cytosolic Na^+^ levels may also increase Ca^2+^ concentration. We have demonstrated that inhibition of the late Na^+^ current reduces increased cellular Na^+^ in HF and secondary diastolic Ca^2+^ which indeed led to a reduced diastolic dysfunction in human HF preparations (Sossalla et al., 2008). This mechanism may theoretically also reduce the arrhythmia potential and could explain the early mortality benefit seen (i.e., less sudden cardiac deaths) in this otherwise high risk population. Thus, distorted Na^+^/Ca^2+^ interplay detrimentally contributes to diastolic dysfunction in HFrEF (Sossalla et al., 2008).

Interestingly, SGLT2i has been recently reported to inhibit the late Na^+^ current in murine HF cardiomyocytes, which constitutes an abnormal Na^+^ influx throughout the action potential (Philippaert et al., 2021). In molecular docking simulations these effects were proposed to be driven by binding of empagliflozin on major cardiac Na^+^ channel isoform Na_*V*_1.5 (Philippaert et al., 2021). While the role of SGLT2i for late Na^+^ current needs to be further investigated in HFpEF, the reduction of late Na^+^ current could favorably impact diastolic function (Sossalla et al., 2011). Interestingly, in patients with hypertrophic cardiomyopathy, typically characterized by normal systolic function but severely disturbed myocardial relaxation, the late Na^+^ current has been demonstrated to deteriorate cardiomyocyte Na^+^ and thus Ca^2+^ balance as a mechanism for impaired diastolic function (Coppini et al., 2013; Ferrantini et al., 2018). On the other hand, in patients with HFpEF and hypertensive heart disease an increased diastolic Ca^2+^ with impaired relaxation was found, which was, however, not caused by elevated Na^+^ levels in this model (Runte et al., 2017). Thus, further studies of cardiomyocyte Na^+^ homeostasis with respect to SGLT2i are needed, in particular in HFpEF myocardium.

Another mechanism by which SGLT2i may influence myocardial Na^+^ homeostasis are inhibitory effects on the Na^+^/H^+^ exchanger 1 (NHE1), which were first reported for rabbit myocardium (Baartscheer et al., 2017), and later confirmed for murine cardiomyocytes (Uthman et al., 2018) and human atrial tissue (Trum et al., 2020). A study in healthy rabbit cardiomyocytes reported a consecutive acute reduction in cytosolic Ca^2+^ and Na^+^ by empagliflozin (Baartscheer et al., 2017). On the contrary the effects on Na^+^ homeostasis via NHE1 inhibition have been questioned in a study using healthy rat cardiomyocytes (Chung et al., 2020).

Finally, overall cytosolic Na^+^ levels were also decreased by empagliflozin after 30 min and 24 h treatment in murine wild-type mice (Mustroph et al., 2018). Therefore, SGLT2i-dependent changes in myocardial Na^+^ might constitute an important cardiac mechanism potentially also in HFpEF (Trum et al., 2021). In conclusion, studies in different experimental models reported an involvement of SGLT2i in cellular Ca^2+^ and Na^+^ homeostasis. Yet, the role of cellular Ca^2+^ and Na^+^ alterations with respect to treatment with SGLT2i in HFpEF is rather speculative and further studies in HFpEF myocardium are required to clarify this important question.

### Inflammation and Oxidative Stress: The Joint Mechanism of Sodium-Glucose-Cotransporter 2 Inhibitors?

**FIGURE 3:**
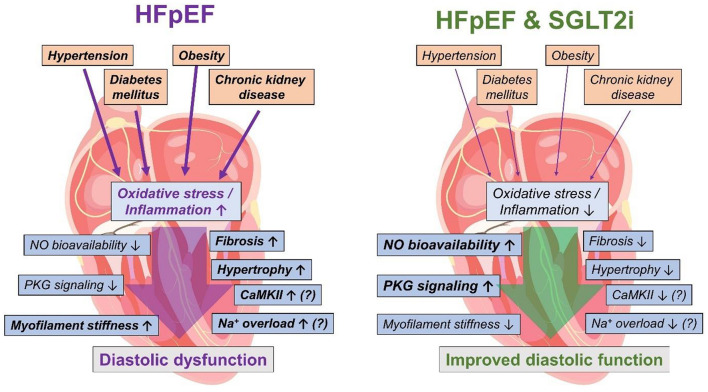
Pathophysiology involved in HFpEF leading to diastolic dysfunction (left) and possible beneficial mechanisms of SGLT2i in HFpEF (right). HFpEF, heart failure with preserved ejection fraction; NO, nitric oxide; PKG, protein kinase G; CaMKII, Ca^2+^/calmodulin-dependent protein kinase IIδ; SGLT2i, sodium-glucose-cotransporter 2 inhibitors. The heart image is licensed by Shutterstock.com/Usama Nasir MD.

In HFpEF, inflammation and oxidative stress play a key role for the progression of structural and functional diastolic dysfunction and are associated with comorbidities typically found in HFpEF patients (Figure 3) such as chronic kidney disease or metabolic syndrome (Franssen et al., 2016; Zhazykbayeva et al., 2020). In particular in cardiorenal syndrome, kidney injury-associated chronic inflammation and oxidative activation may impair cardiac function as shown in different models of renal failure (Rangaswami et al., 2019). Thus, oxidative stress and inflammation are considered as central mechanisms linking the cardiac HFpEF phenotype with the multifaceted comorbidities in the HFpEF patient (Zhazykbayeva et al., 2020). Growing evidence demonstrates that SGLT2i attenuate inflammation and oxidative stress (Yaribeygi et al., 2019). In diabetic mice with myocardial infarction SGLT2i reduced oxidative stress and inflammatory markers (Ye et al., 2017; Yurista et al., 2019). Likewise, 4 weeks of treatment with ipragliflozin diminished oxidative stress and inflammation in diabetic mice (Tahara et al., 2013, 2014).

In HFpEF, also inflammation/oxidative stress-mediated endothelial dysfunction may impair cardiomyocyte function (Franssen et al., 2016). A recent work demonstrated that empagliflozin may reduce inflammation-dependent endothelial dysfunction resulting in improved cardiomyocyte contractility (Juni et al., 2019). In line with that, dapagliflozin reduced endothelial dysfunction and inflammation in a HFpEF rat model (Dahl salt-sensitive rats with high-salt diet) resulting in an improved diastolic function (Cappetta et al., 2020). Moreover, we showed that empagliflozin attenuated pathologically elevated levels of oxidative stress (H_2_O_2_, GSH, LPO) and inflammation (ICAM, VCAM, TNFα, and IL-6) in human HFpEF myocardium after *in vitro* treatment (Kolijn et al., 2021).

As empagliflozin reduced oxidative stress and inflammation in human HFpEF myocardium, NO bioavailability and PKG signaling were improved upon exposure to empagliflozin leading to lower myofilament stiffness and thereby improved diastolic function in human myocardium (Pabel et al., 2018; Kolijn et al., 2021). Thus, the attenuation of oxidative stress and inflammation due to SGLT2i treatment could be potentially helpful in HFpEF patients (Figure 3) at least via an improvement of contractility (Pabel et al., 2020a). Also, other potential secondary effects of SGLT2i driven by a reduction of oxidative stress and inflammation are conceivable. A potential oxidative CaMKII activation (Erickson et al., 2008) might be diminished as SGLT2i reduce oxidative stress, which could result in lower diastolic sarcoplasmic reticulum Ca^2+^ leak as well as reduced late Na^+^ current (Mustroph et al., 2018; Philippaert et al., 2021). Finally, hypertrophy and fibrosis are a common detrimental outcome of chronic inflammation and oxidative stress, and could thereby be ameliorated upon anti-oxidative and anti-inflammatory effects of SGLT2i (Zhazykbayeva et al., 2020). However, the molecular mechanisms need to be explored further.

## Conclusion

As we are on the cusp of welcoming the first prognostically beneficial drug class in HFpEF, understanding the mechanistic effects of SGLT2i on the myocardium will be key in maximizing its potential in this important patient population. While some putative targets and pathways are still rather speculative, evidence from human myocardium including human HFpEF hearts indicate direct favorable effects on diastolic function via reduced myofilament stiffness due to improved PKG signaling. While this review discusses some potentially relevant mechanisms of SGLT2i in HFpEF, also other pleiotropic effects of SGLT2i have been described as discussed elsewhere (Packer, 2020). Both the EMPEROR-Preserved and the DELIVER trials will, undoubtedly, provide further insight into the extent to which SGLT2i will have an impact on the treatment of HFpEF patients in the near future.

## Author Contributions

SP, NH, JS, and SS drafted and revised the manuscript. All authors contributed to the article and approved the submitted version.

## Conflict of Interest

SP received speaker’s honoraria from AstraZeneca. JS has received speaker’s honoraria from Boehringer Ingelheim Pharma GmbH and AstraZeneca. SS received speaker’s/consultancy honoraria from Boehringer Ingelheim Pharma GmbH and AstraZeneca. The remaining author declares that the research was conducted in the absence of any commercial or financial relationships that could be construed as a potential conflict of interest.

## Publisher’s Note

All claims expressed in this article are solely those of the authors and do not necessarily represent those of their affiliated organizations, or those of the publisher, the editors and the reviewers. Any product that may be evaluated in this article, or claim that may be made by its manufacturer, is not guaranteed or endorsed by the publisher.
